# Letter to the editor regarding the Paper “S2k-Guideline hand antisepsis and hand hygiene”

**DOI:** 10.3205/dgkh000637

**Published:** 2026-03-02

**Authors:** Ralf Schäfer

**Affiliations:** 1 Mölnlycke Health Care GmbH, Düsseldorf, Germany

## Letter to the Editor

Dear Editor,

I read with great interest your 2024 update of the *S2k Guideline on Hand Antisepsis and Hand Hygiene* published in GMS Hygiene and Infection Control [1]. The guideline rightly emphasizes the high frequency of glove perforations as a relevant risk factor for nosocomial infections and consequently recommends double gloving during procedures with elevated exposure risk.

In this context, I would like to highlight an important conceptual distinction that may warrant further consideration: the difference between standard double gloving and the use of indicator glove systems.

Evidence from systematic reviews and meta-analyses shows that double gloving significantly improves protection of the inner glove layer and enhances self-protection for surgical staff, particularly in high-risk surgical procedures [2]. However, double gloving alone does not fundamentally prevent glove perforations; it primarily reduces the likelihood that perforations reach the inner glove.

Indicator glove systems serve a different purpose. Their primary mechanism is rapid visual detection of perforations via color contrast rather than enhanced physical resistance. A systematic review and metasynthesis [3] demonstrated substantially improved perforation detection: while detection under standard double glove conditions reaches around 34%, indicator systems achieve rates exceeding 80%. This constitutes a distinct safety benefit through timely recognition and glove change, rather than through barrier reinforcement.

In this light, I would like to comment on Article 47 of your guideline. The statement “The number of perforations of the inner glove during surgery was not significantly lower when indicator gloves were worn compared with standard” appears to conflate two separate dimensions: protective efficacy (inner glove perforation rates), relevant to double gloving, and detection efficacy, which is the primary advantage of indicator systems. Since indicator gloves are not designed to reduce inner glove perforations but to enhance visibility of perforations, the comparison may inadvertently obscure their distinct value in intraoperative safety. 

Thank you for your important contribution to advancing hygiene and infection prevention. I would welcome your expert perspective on this matter.

## Notes

### Competing interests

The author is employed as Key Account Manager Gloves at Mölnlycke Health Care GmbH.

### Funding

No external funding was received for this work.
